# Effect of Simulated Oral Aging on Surface Roughness and Microhardness of Bulk-Fill Composite Resins

**DOI:** 10.3390/dj14060366

**Published:** 2026-06-15

**Authors:** Alexandru Mihai Tănasă, Ionuț Tărăboanță, Irina Nica, Andra Claudia Tărăboanță-Gamen, Nicoleta Tofan, Răzvan Constantin Brânzan, Corina Alexandra Brânză-Concită, Sorin Andrian

**Affiliations:** Grigore T. Popa University of Medicine and Pharmacy, 16 Universitatii Street, 700115 Iasi, Romania; alexandru-mihai.tanasa@umfiasi.ro (A.M.T.); nica.irina@umfiasi.ro (I.N.); andra-claudia.gamen@umfiasi.ro (A.C.T.-G.); nicoleta.tofan@umfiasi.ro (N.T.); razvan-constantin.branzan@umfiasi.ro (R.C.B.); corina-alexandra.concita@umfiasi.ro (C.A.B.-C.); sorin.andrian@umfiasi.ro (S.A.)

**Keywords:** bulk-fill resin composites, artificial aging, pH-cycling, thermocycling, mechanical wear, surface roughness, Vickers microhardness, dental composites

## Abstract

**Introduction:** The goal of this study was to evaluate the influence of combined artificial aging protocols on the surface roughness and Vickers microhardness of bulk-fill resin composites, compared with a nanofilled composite used as a reference. **Materials and Methods:** A total of 120 cylindrical specimens were prepared from three bulk-fill composites (Tetric EvoCeram Bulk Fill, Filtek One Bulk Fill, Venus Bulk Fill) and one nanofilled composite (Filtek Supreme Ultra). Specimens were allocated into three aging conditions: mechanical wear (A), mechanical wear combined with pH-cycling (B), and mechanical wear combined with thermocycling (C). Surface roughness (Ra) and Vickers microhardness (VHN) were evaluated at two time points (T1: 120,000 cycles; T2: 240,000 cycles). Non-parametric statistical tests were applied (α = 0.05). **Results:** Aging protocols significantly influenced both Ra and VHN (*p* < 0.05). Overall, higher surface roughness and lower Vickers microhardness values were observed after cumulative aging, with material-dependent variations between T1 and T2. The greatest post-aging differences were observed under combined mechanical wear and pH-cycling (subgroup B), whereas mechanical wear alone showed the lowest changes. Filtek One Bulk Fill and Filtek Supreme Ultra showed more favorable post-aging Ra and VHN values, whereas Venus Bulk Fill showed less favorable post-aging surface properties. No significant correlation was found between Ra and VHN (rho = −0.009; *p* = 0.958). **Conclusions:** Combined aging conditions significantly affected the surface roughness and Vickers microhardness of resin composites, with the greatest post-aging differences observed under acidic challenges. Bulk-fill materials exhibit variable resistance depending on composition, emphasizing the importance of material selection for long-term clinical performance. **Clinical relevance:** Composite restorations exposed to combined mechanical and acidic challenges may show altered surface roughness and microhardness, highlighting the need for materials with enhanced resistance in high-risk oral environments.

## 1. Introduction

Resin-based composite materials have become the cornerstone of contemporary restorative dentistry due to their ability to simultaneously fulfill functional and aesthetic requirements [1]. Continuous advancements in filler technology, resin matrices, and photoinitiator systems have significantly improved their mechanical performance and clinical longevity, thereby expanding their indications in both anterior and posterior restorations [2].

Bulk-fill resin composites have been developed to simplify restorative procedures by allowing placement in thicker increments, typically up to 4–5 mm, while maintaining an adequate degree of conversion throughout the material [3,4]. This performance is achieved through modifications in translucency, photoinitiator systems, and polymer network structure, which enhance light penetration and polymerization efficiency [5]. However, these structural modifications may also influence the long-term mechanical stability of the materials, particularly under clinically relevant aging conditions [6,7,8].

In the oral environment, restorative materials are exposed to a complex combination of mechanical, chemical, and thermal challenges. Mechanical stresses generated by mastication contribute to fatigue and wear, while chemical degradation induced by dietary acids and bacterial metabolism may affect the organic matrix and the filler–matrix interface [5,9,10]. In addition, temperature fluctuations lead to cyclic stresses due to differences in thermal expansion between material components [11]. These factors rarely act independently, and their combined effects are considered responsible for the long-term degradation of resin-based materials in vivo [12].

Artificial aging protocols such as thermocycling, pH-cycling, and mechanical wear simulation have been widely used to reproduce specific aspects of the oral environment [13,14]. However, most studies have evaluated these factors in isolation, while their combined effects—more representative of clinical conditions—remain insufficiently explored. This limitation restricts the ability to accurately predict the long-term clinical behavior of bulk-fill composites under complex oral challenges [15].

Furthermore, although nanofilled composites are often considered a reference standard due to their favorable balance between mechanical strength, wear resistance, and surface stability [16], comparative data between these materials and bulk-fill composites under multifactorial aging conditions remain limited.

Therefore, the aim of the present in vitro study was to evaluate the influence of combined artificial aging protocols based on mechanical wear, either applied alone or in association with pH-cycling or thermocycling, on the surface roughness and Vickers microhardness of selected bulk-fill resin composites, in comparison with a nanofilled composite used as a control. Surface roughness and Vickers microhardness were selected as clinically relevant indicators of post-aging surface condition and material performance under simulated oral challenges.

The null hypotheses tested were as follows: (1) no significant differences exist in surface roughness and microhardness among the investigated materials; (2) the applied aging protocols do not significantly affect the investigated properties; and (3) no significant changes occur over time between the two evaluation intervals.

## 2. Materials and Methods

### 2.1. Study Design

The present study was designed as an in vitro experimental investigation to evaluate post-aging surface roughness and microhardness values of resin-based restorative materials under simulated oral aging conditions. A multifactorial design was employed, considering material type and aging protocol as independent variables, and surface roughness (Ra) and Vickers microhardness (VHN) as dependent variables.

The experimental model was developed to reproduce clinically relevant oral challenges by combining mechanical wear with either chemical (pH-cycling) or thermal (thermocycling) stresses. Mechanical wear was applied as a baseline condition for all specimens, while additional aging factors were superimposed to simulate more complex degradation scenarios.

All specimens were evaluated at two predefined time points (T1 and T2), allowing a longitudinal assessment of material behavior. The same specimens were evaluated at both time points. To minimize measurement-induced interference, T1 and T2 measurements were performed on predefined, spatially separated, non-overlapping regions of the exposed surface. The aging procedures were applied to the entire specimen surface; therefore, T2 values reflect material behavior after a longer cumulative exposure period, whereas the use of separate measurement regions was intended only to avoid direct overlap with the areas analyzed at T1.

All procedures, including specimen preparation, aging protocols, and testing methods, were performed under standardized laboratory conditions to ensure reproducibility and minimize experimental variability.

The study was conducted in accordance with the ethical principles outlined in the Declaration of Helsinki and received approval from the Ethics Committee of “Grigore T. Popa” University of Medicine and Pharmacy, Iași, Romania (approval no. 616/30.06.2025).

### 2.2. Materials

The materials included in the present study consisted of three bulk-fill resin composites and one nanofilled composite used as a control reference. The investigated bulk-fill materials were Tetric EvoCeram Bulk Fill (Ivoclar Vivadent), Filtek One Bulk Fill Restorative (3M ESPE), and Venus Bulk Fill (Kulzer), while the nanofilled composite Filtek Supreme Ultra (3M ESPE) was used as the control material.

Material selection was based on their widespread clinical use and differences in composition, particularly with regard to resin matrix formulation, filler type, and filler loading, which are known to influence mechanical performance and resistance to degradation.

All materials were handled according to the manufacturers’ instructions. For standardization purposes, a single shade was selected for each composite, and all specimens were prepared under identical environmental conditions.

Light polymerization was performed using an LED curing unit (Bluephase N^®^, Ivoclar Vivadent, Schaan, Liechtenstein), operating within a wavelength range of 385–515 nm. The light intensity was maintained at approximately 1200 mW/cm^2^, and curing time was applied according to the manufacturers’ recommendations. The curing tip was positioned perpendicular to the specimen surface and kept in direct contact with the Mylar strip to ensure a consistent curing distance. The output of the curing unit was periodically verified using a calibrated radiometer (Bluephase Meter II^®^, Ivoclar Vivadent).

The detailed composition of the investigated materials, including organic matrix components, filler characteristics, and filler loading (wt% and vol%), is presented in Table 1.

All materials were stored under conditions recommended by the manufacturers prior to use, and no material was used beyond its expiration date.

### 2.3. Sample Preparation

Sample size calculation was performed using G*Power 3.1 (Heinrich-Heine University, Düsseldorf, Germany), assuming a significance level of 0.05, a statistical power of 80%, and a moderate-to-large effect size (f = 0.40) derived from a preliminary pilot study. This resulted in a minimum required sample of 72 specimens. Therefore, a total of 120 specimens was considered sufficient to ensure adequate statistical power and balanced group allocation.

A total of 120 cylindrical specimens were fabricated from the investigated resin-based composites, including three bulk-fill materials, Tetric EvoCeram Bulk Fill (Group I), Filtek One Bulk Fill Restorative (Group II), and Venus Bulk Fill (Group III), and one nanofilled composite used as a control, Filtek Supreme Ultra (Group IV). Each group consisted of 30 specimens, which were further subdivided into three subgroups (A–C) according to the applied aging protocol (n = 10 per subgroup). The distribution of the samples in groups and subgroups is presented below:

Group I—Tetric EvoCeram Bulk Fill: 30 specimens

- Subgroup IA: mechanical wear, n = 10.
- Subgroup IB: mechanical wear combined with pH-cycling, n = 10.
- Subgroup IC: mechanical wear combined with thermocycling, n = 10.

Group II—Filtek One Bulk Fill Restorative: 30 specimens

- Subgroup IIA: mechanical wear, n = 10.
- Subgroup IIB: mechanical wear combined with pH-cycling, n = 10.
- Subgroup IIC: mechanical wear combined with thermocycling, n = 10.

Group III—Venus Bulk Fill: 30 specimens

- Subgroup IIIA: mechanical wear, n = 10.
- Subgroup IIIB: mechanical wear combined with pH-cycling, n = 10.
- Subgroup IIIC: mechanical wear combined with thermocycling, n = 10.

Group IV—Filtek Supreme Ultra: 30 specimens

- Subgroup IVA: mechanical wear, n = 10.
- Subgroup IVB: mechanical wear combined with pH-cycling, n = 10.
- Subgroup IVC: mechanical wear combined with thermocycling, n = 10.

Total: 120 specimens.

All specimens were prepared in a standardized cylindrical geometry, with a diameter of 6 mm and a thickness of 6 mm. Bulk-fill materials were inserted in a single increment, while the nanofilled composite was placed in two increments of 3 mm. A polyester (Mylar) strip and a glass slide were applied to the top surface, and a standardized pressure was used to extrude excess material and minimize air entrapment.

After polymerization, the composite cylinders were embedded in autopolymerizing acrylic resin blocks, leaving an exposed composite portion of approximately 3 mm for aging and surface testing, while the remaining portion was embedded in acrylic resin to ensure specimen stabilization during the experimental procedures.

Light polymerization was performed using an LED curing unit (Bluephase N^®^, Ivoclar Vivadent), delivering an intensity of approximately 1200 mW/cm^2^. The curing tip was positioned perpendicular to the specimen surface and maintained in direct contact with the Mylar strip. Light output was periodically verified using a calibrated radiometer to ensure consistency.

After polymerization, specimens were removed from the molds and visually inspected under magnification. Specimens presenting defects such as porosities or surface irregularities were discarded and replaced. Subsequently, the composite cylinders were embedded in autopolymerizing acrylic resin blocks (Duracryl^®^, SpofaDental, Kralovehradecky, Czech Republic), leaving an exposed composite surface of approximately 3 mm for further testing.

The exposed composite surfaces were finished and polished using a standardized sequence of aluminum oxide abrasive discs (Sof-Lex™, 3M ESPE, Mumbai, India), comprising medium (beige) and fine (white) grits, under continuous water cooling, to obtain comparable surface characteristics across all specimens.

Following finishing procedures, all specimens were stored in distilled water at 37 °C for 24 h to allow post-polymerization prior to aging.

Each specimen was subsequently allocated to a specific aging protocol (subgroups A–C), while the same specimens were evaluated at both time points (T1 and T2) using predefined, non-overlapping surface regions.

All preparation procedures were carried out by a single operator under controlled environmental conditions to minimize variability and enhance reproducibility.

**Figure 1:**
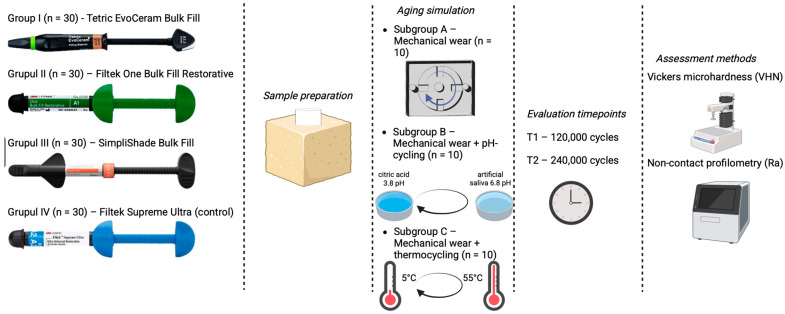
Illustration of the study design.

The study design is illustrated in Figure 1.

### 2.4. Aging Procedures

The artificial aging protocols were designed to simulate, under controlled laboratory conditions, the combined mechanical, chemical, and thermal challenges encountered in the oral environment. In this experimental model, chemical degradation was reproduced through pH-cycling, while thermal stress was simulated by thermocycling. Mechanical wear was applied as the primary factor in all experimental groups.

In the combined aging groups, pH-cycling or thermocycling was performed simultaneously with mechanical wear throughout the entire wear simulation. These procedures were not applied after completion of mechanical loading, but were synchronized with the wear protocol to reproduce combined mechanical–chemical or mechanical–thermal challenges under standardized laboratory conditions.

#### 2.4.1. Mechanical Wear Simulation

Mechanical wear was performed using a custom-designed chewing simulator developed to reproduce masticatory dynamics under standardized conditions. The system was based on the “Rub&Roll” device principle [17], which involves cyclic sliding and rolling contact under constant load, a mechanism commonly employed in in vitro wear simulation studies.

Each acrylic block containing the specimen was mounted on a rotating platform and subjected to contact with two antagonistic rods coated with polyvinyl chloride (PVC). A constant load of 50 N was applied, with a rotational speed of 20 rpm. The wear simulation was carried out in two intervals: 120,000 cycles (T1) and 240,000 cycles (T2).

The numbers for mechanical loading cycles were selected to represent two progressive stages of simulated oral aging. The first evaluation point, 120,000 cycles, was intended to reflect an early aging interval, whereas 240,000 cycles represented a more advanced degradation stage. Although a direct conversion between chewing-simulator cycles and clinical service time is not universally standardized, an approximate interpretation can be made using commonly cited estimates of average masticatory frequency. Based on an estimated 240,000 chewing cycles per year, 120,000 cycles may correspond roughly to 6 months of clinical function, while 240,000 cycles may approximate 1 year of function. These values should be interpreted only as approximate clinical equivalents, since actual masticatory frequency varies considerably among individuals and is influenced by diet, parafunctional habits, occlusal conditions, and restoration location. Therefore, the selected intervals were used primarily as controlled comparative aging points rather than exact simulations of clinical service time.

The device allowed precise control of load, speed, and number of cycles, ensuring reproducible testing conditions across all specimens.

#### 2.4.2. pH-Cycling Protocol

For specimens assigned to subgroup B, mechanical wear was combined with a pH-cycling protocol designed to simulate fluctuations between demineralizing and remineralizing conditions.

The pH-cycling protocol was adapted from previously described in vitro erosive challenge models [18]. Specimens were alternately immersed in 0.3% citric acid solution, adjusted to pH 3.8 and artificial saliva (pH 6.8). The artificial saliva contained NaCl 0.400 g/L, KCl 0.400 g/L, CaCl_2_·2H_2_O 0.906 g/L, NaH_2_PO_4_·2H_2_O 0.690 g/L, Na_2_S·9H_2_O 0.005 g/L, and urea 1.000 g/L in distilled water. One pH cycle consisted of 30 s immersion in citric acid, a 15 s transition period, 30 s immersion in artificial saliva, and a 15 s transition period before the next cycle. This sequence was repeated continuously during the mechanical wear simulation. The solutions were monitored and renewed daily to maintain stable pH and composition. Based on this sequence, 3840 pH cycles were completed up to T1 and 7680 cycles up to T2.

The 30 s citric acid exposure was selected to simulate short, repeated erosive episodes associated with acidic dietary intake. Although longer exposure times have been used in previous in vitro studies, the present protocol was applied continuously during mechanical wear and was intended as an accelerated, standardized erosive challenge rather than a direct reproduction of clinical acid exposure.

#### 2.4.3. Thermocycling Protocol

Specimens in subgroup C were subjected to thermocycling in addition to mechanical wear. Thermocycling was performed within the temperature range between 5 °C and 55 °C. Each thermal cycle consisted of immersion in the 5 °C bath for 30 s and in the 55 °C bath for 30 s, with a transfer time of 15 s between baths. Thermocycling was synchronized with the mechanical wear protocol and applied throughout the experimental aging period. A total of 10.000 thermal cycles were completed up to T1, corresponding to 120,000 mechanical wear cycles, and 20.000 thermal cycles were completed up to T2, corresponding to 240,000 mechanical wear cycles.

The pH-cycling and thermocycling procedures were applied continuously during mechanical wear simulation in order to create an accelerated aging model and to enhance the ability to detect material-dependent differences within a controlled experimental timeframe. This approach does not aim to reproduce the exact intermittent nature of acidic and thermal challenges in vivo, where salivary clearance, pellicle formation, dietary habits, and individual behavioral factors modulate exposure. Instead, the protocol was designed as a standardized worst-case simulation of combined mechanical, chemical, and thermal stresses.

#### 2.4.4. Experimental Timeline

All specimens were evaluated at two predefined time points:

T1—after 120,000 mechanical loading cycles, corresponding to the first accelerated aging interval.

T2—after 240,000 mechanical loading cycles, corresponding to the second accelerated aging interval.

The 4- and 8-day durations refer exclusively to the laboratory time required to complete the accelerated aging protocols and should not be interpreted as direct clinical exposure periods. When considering an approximate estimate of 240,000 masticatory cycles per year, the two mechanical loading intervals may be interpreted as corresponding roughly to 6 months and 1 year of clinical function, respectively; however, these estimates remain approximate and should be considered only for contextual interpretation.

This design enabled the assessment of both early and progressive changes in material behavior under simulated aging conditions.

### 2.5. Mechanical Properties and Surface Analysis

#### 2.5.1. Surface Profilometry (2D Non-Contact Analysis)

Surface alterations induced by the applied aging protocols were assessed using a non-contact profilometer (Dektak XT, Bruker, Billerica, MA, USA). Measurements were performed on standardized regions of the exposed composite surfaces.

The same specimens were evaluated at both time points (T1 and T2). In order to avoid interference between repeated measurements, the exposed surface of each specimen was divided into two distinct, non-overlapping regions by means of a fine reference line drawn at the midpoint of the surface. One region was allocated for T1 evaluation, while the opposite region was reserved for T2 assessment.

The two surface regions were defined in a standardized manner on each specimen to minimize measurement overlap and operator-related variability. Although specimens were prepared, finished, and polished under identical conditions, baseline equivalence between the two regions was not assessed separately.

Profilometric analysis was carried out using a cut-off value of 0.4 mm and a scanning length of 4 mm. Ten scans were performed in intersecting directions for each specimen. Additionally, three independent measurements were recorded per sample, with a 120° rotation between determinations, and the final arithmetic mean roughness (Ra) was calculated.

All measurements were conducted under standardized environmental conditions, with consistent specimen positioning and prior calibration of the instrument according to the manufacturer’s instructions.

#### 2.5.2. Vickers Microhardness

Vickers microhardness and elastic modulus were evaluated using a tribometer (CETR UMT-2, Bruker Corporation, Berlin, Germany), equipped with dedicated software. Measurements were performed by instrumented indentation using a diamond indenter with a square-based pyramidal geometry (136° tip angle). Hardness values were automatically calculated based on the applied load and penetration depth.

A load of 9.8 N (1 kgf) was applied for 15 s, and the results were expressed as Vickers hardness number (VHN). The applied force and penetration depth were continuously recorded during the indentation process using integrated sensors.

The same specimens were evaluated at both time points (T1 and T2). To prevent interference between measurements, the exposed surface of each specimen was divided into two distinct regions using a fine reference line drawn at the midpoint. One region was used exclusively for T1 evaluation, while the other region was reserved for T2. Both regions were predefined using the same standardized approach; however, separate baseline equivalence between the T1 and T2 indentation areas was not assessed.

For each time point, three indentations were performed per specimen within the allocated region, maintaining a minimum distance of 2 mm between indentation sites to avoid interaction effects. The mean value was used for statistical analysis.

Vickers indentations performed at T1 were localized within the region allocated exclusively to the first evaluation interval. For T2, indentations were performed in a distinct non-overlapping region, separated from the T1 indentation sites by the reference line. This procedure was used to avoid direct mechanical interference from previous indentation marks. However, because the same specimens were subjected to continued aging between T1 and T2, the later measurements represent cumulative material degradation rather than a fully independent surface condition.

### 2.6. Statistical Analysis

Statistical analysis was performed using SPSS Statistics (version 29.0.0; IBM Corp., Armonk, NY, USA).

Data distribution was assessed using the Shapiro–Wilk test, which indicated a non-normal distribution for the majority of variables; therefore, non-parametric statistical methods were applied.

Comparisons between independent groups (materials and aging protocols) were performed using the Kruskal–Wallis test. When statistically significant differences were identified, pairwise comparisons were conducted using the Dunn–Bonferroni post hoc test.

Comparisons between T1 and T2 were performed using the Wilcoxon signed-rank test, as measurements were obtained from the same specimens at different time points, although in predefined non-overlapping surface regions.

Correlation between surface roughness (Ra) and Vickers microhardness (VHN) was assessed using Spearman’s rank correlation coefficient.

The level of statistical significance was set at *p* < 0.05.

## 3. Results

### 3.1. Descriptive Statistics of the Evaluated Surface Properties

Vickers microhardness varied according to both material type and aging protocol. Filtek Supreme Ultra and Filtek One Bulk Fill generally maintained the highest VHN values, whereas Venus Bulk Fill showed the lowest values across most conditions. The greatest reduction in microhardness was observed when mechanical wear was combined with pH-cycling, while thermocycling produced intermediate changes. A slight decrease from T1 to T2 was observed in most groups, indicating progressive aging-related degradation. The complete descriptive values are presented in Table 2.

Surface roughness showed a progressive increase after simulated aging, with higher Ra values generally recorded at T2 than at T1. The most pronounced roughness changes were observed under mechanical wear combined with pH-cycling, followed by thermocycling, while mechanical wear alone produced lower surface alteration. Venus Bulk Fill tended to show higher Ra values, whereas Filtek One Bulk Fill and Filtek Supreme Ultra generally presented lower roughness values. The complete descriptive values are presented in Table 3.

### 3.2. Surface Roughness (Ra)

Surface roughness showed a general tendency toward higher post-aging values, particularly after combined mechanical wear and pH-cycling; however, statistically significant T1–T2 increases were observed only in selected material–protocol combinations. At T1, the lowest Ra values were observed in subgroup A (mechanical wear), while the highest values were recorded in subgroup B (mechanical wear combined with pH-cycling). A similar pattern was observed at T2, although the magnitude and statistical significance of T1–T2 changes varied according to material and aging protocol.

Across all experimental conditions, a consistent ranking of materials was observed, with Group II and Group IV exhibiting lower roughness values, while Group III showed the highest Ra values.

Statistically significant differences in surface roughness were identified among materials within each subgroup at both time points (*p* < 0.05). Pairwise comparisons confirmed that Group III differed significantly from most other materials, whereas differences between Groups II and IV were generally not significant.

The aging protocol had a significant effect on surface roughness (*p* < 0.05). Subgroup B consistently exhibited the highest Ra values, indicating the most pronounced surface degradation, followed by subgroup C, while subgroup A showed the lowest roughness values.

A general increase in Ra was observed from T1 to T2, with statistically significant time-dependent changes detected in the overall analysis (*p* < 0.05). The greatest changes were recorded in subgroup B.

**Figure 2:**
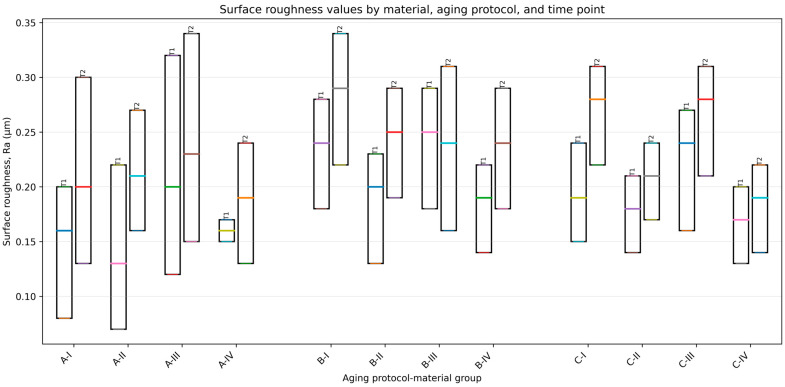
Boxplot representation of surface roughness values (Ra) for all tested materials (Groups I–IV) under different aging protocols (A–C) at both evaluation time points (T1 and T2). Boxes indicate the interquartile range, and horizontal lines indicate medians. Group codes: I—Tetric EvoCeram Bulk Fill; II—Filtek One Bulk Fill Restorative; III—Venus Bulk Fill; IV—Filtek Supreme Ultra.

The distribution of Ra values is presented in Figure 2 as boxplot/dot plot graphs, while representative profilometric profiles are provided in Supplementary Figure S1.

To further explore the differences in surface roughness among the investigated materials, pairwise comparisons were performed using the Dunn–Bonferroni post hoc test. The analysis was conducted separately for each aging protocol and evaluation time point. The results are presented in Table 4.

Representative profilometric profiles are provided in Supplementary Figure S1. These profiles were selected as illustrative examples of the surface topography observed at T2 under the three aging protocols. They are intended to complement the quantitative Ra data presented in Figure 2 and Table 3 and should not be interpreted as additional quantitative measurements.

The profilometric profiles in Supplementary Figure S1 visually illustrate typical surface patterns observed in groups with different Ra values. More irregular profiles were generally observed in groups with higher Ra values, particularly after combined mechanical wear and pH-cycling, whereas smoother profiles were observed in groups with lower Ra values. These profiles should be interpreted only as illustrative examples.

### 3.3. Vickers Microhardness

Vickers microhardness values were generally lower after more intensive aging conditions, particularly in subgroup B. Across the evaluated aging protocols and time points, the highest median VHN values were recorded for Group IV, followed by Group II, Group I, and Group III. However, post hoc comparisons showed that differences between Groups II and IV were generally not statistically significant, indicating comparable post-aging microhardness behavior between these two materials.

Subgroup B (mechanical wear combined with pH-cycling) generally exhibited the lowest post-aging microhardness values, whereas subgroup A showed the highest values. Subgroup C presented intermediate results.

Statistically significant differences in microhardness were identified among materials within all subgroups at both time points (*p* < 0.05). Group II and Group IV consistently demonstrated higher VHN values compared to Groups I and III, while Group III showed the lowest resistance to degradation.

The aging protocol significantly influenced microhardness values (*p* < 0.05), with subgroup B showing the most pronounced reduction, followed by subgroup C, and subgroup A exhibiting the smallest decrease.

VHN values showed a descriptive decrease from T1 to T2 in several material–protocol combinations. However, within-group Wilcoxon signed-rank analysis showed statistically significant decreases only for Group I in subgroups A and B (*p* < 0.05), whereas the remaining comparisons were not statistically significant (*p* > 0.05).

**Figure 3:**
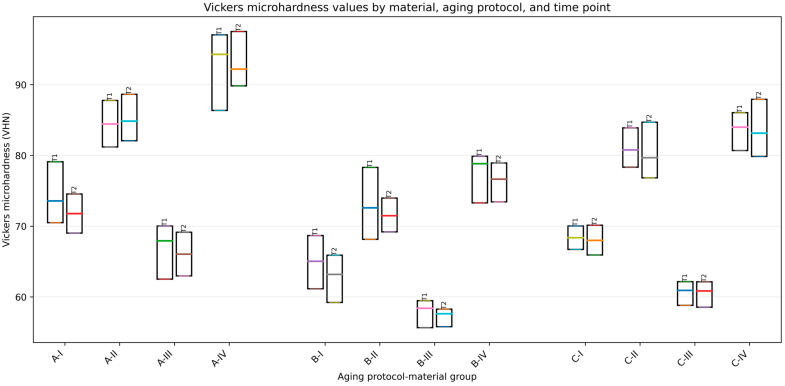
Boxplot/dot plot representation of Vickers microhardness values (VHN) for all tested materials (Groups I–IV) under different aging protocols (A–C) at both evaluation time points (T1 and T2). Boxes indicate the interquartile range, horizontal lines indicate medians, and dots represent individual values.

The distribution of VHN values is illustrated in Figure 3 as boxplot/dot plot graphs and detailed pairwise comparisons are presented in Table 5 (Figure S1).

The results of the pairwise comparisons between groups, based on the Dunn–Bonferroni post hoc test, are presented in Table 5.

### 3.4. Within-Group Comparisons Between T1 and T2

Within-group comparisons between T1 and T2 were performed using the Wilcoxon signed-rank test. For surface roughness, statistically significant increases were observed mainly in subgroup B for Groups I, II, and IV, and in subgroup C for Group I (*p* < 0.05), while the remaining comparisons were not significant (*p* > 0.05). For Vickers microhardness, a statistically significant decrease was observed for Group I in subgroups A and B (*p* < 0.05), whereas the other material–protocol combinations showed non-significant changes over time (*p* > 0.05). Therefore, non-significant T1–T2 variations were interpreted as descriptive trends rather than statistically supported changes.

### 3.5. Correlation Analysis Between Microhardness (VHN) and Surface Roughness (Ra)

Spearman correlation analysis revealed no statistically significant association between surface roughness (Ra) and Vickers microhardness (VHN) across the investigated conditions (rho = −0.009; *p* = 0.958). Within individual subgroups, correlation coefficients ranged from weak positive to weak negative values (subgroup A: rho = −0.009; *p* = 0.958; subgroup B: rho = 0.052; *p* = 0.751; subgroup C: rho = −0.282; *p* = 0.078); however, none of these associations reached statistical significance (*p* > 0.05).

## 4. Discussion

The results of the present study support the rejection of the null hypotheses related to material-dependent differences and the effect of aging protocols on the evaluated surface properties. Significant intergroup differences were identified for both surface roughness and Vickers microhardness; the tested aging protocols were associated with differences in post-aging Ra and VHN values, with material- and protocol-dependent patterns. This pattern is consistent with recent literature demonstrating that thermo-chemical aging can increase surface roughness and reduce microhardness in most composite materials, reflecting deterioration of the superficial layer and weakening of the polymer network. For instance, significant increases in Ra and reductions in microhardness have been reported following erosive challenges combined with thermocycling [18]. Similarly, studies evaluating the combined effects of finishing/polishing procedures and aging protocols (water storage, thermocycling, UV exposure) confirm that material composition remains a major determinant, and that certain bulk-fill composites may perform comparably to reference materials under standardized conditions [19].

Differences between materials can be plausibly explained by matrix composition and filler characteristics, which are recognized determinants of hardness and wear behavior. In the present study, Group III (Venus Bulk Fill) consistently exhibited lower VHN and higher Ra values compared to the other materials, whereas Groups II (Filtek One Bulk Fill) and IV (Filtek Supreme Ultra) showed more favorable and often comparable results, suggesting enhanced resistance to degradation. This hierarchy is consistent with the documented relationship between filler content and microhardness, as VHN is generally more dependent on material composition than on secondary protocol parameters, with filler weight percentage exerting a stronger influence than volumetric fraction [20]. Moreover, state-of-the-art perspectives emphasize that reduced filler size and optimized filler–matrix interfaces are critical for maintaining polishability and wear resistance, while matrices more susceptible to sorption and plasticization tend to degrade over time [21].

The lower VHN and higher Ra values generally observed in subgroup B may indicate that the combination of mechanical wear and pH-cycling produced more pronounced surface alterations under the present experimental conditions. Based on previous literature, acidic exposure may contribute to matrix softening, hydrolytic changes, and weakening of the filler–matrix interface, which could make the surface more susceptible to mechanical wear. However, because no microstructural analysis was performed, filler particle plucking or interfacial degradation cannot be directly confirmed in the present study. This mechanism is supported by recent studies showing that erosive aging combined with thermocycling leads to increased roughness and decreased microhardness, associated with microcracks and porosity due to filler leaching [22,23,24]. Additionally, exposure to acidic environments, such as dietary acids (e.g., orange juice), has been associated with significant reductions in microhardness over time, even when subsequent abrasion has a limited effect [22]. Discrepancies with studies reporting minimal effects of pH-cycling on Ra may be explained by differences in acid type, exposure duration, sequence of challenges, and material susceptibility [25].

The intermediate post-aging values observed in subgroup C may be associated with hydrothermal stresses induced by thermocycling. Cyclic temperature changes and water exposure may contribute to interfacial stress and surface alterations, as suggested by previous studies. Nevertheless, the present study did not include microscopic evaluation; therefore, microdefects or interfacial damage remain possible explanations rather than directly demonstrated findings. The literature supports that thermocycling can significantly reduce microhardness in bulk-fill and nanohybrid composites, with the magnitude of the effect depending on material composition, number of cycles, and conditioning environment [24]. At the mechanistic level, the durability of glass- and ceramic-filled composites is strongly influenced by the silane-mediated interface, where hydrolysis and rearrangement of Si–O–Si bonds may compromise stress transfer and surface integrity [26,27]. Furthermore, classical data on hydrolytic degradation indicate that filler leaching and interfacial degradation may lead to microcrack formation within the matrix, depending on filler composition [28].

Descriptive differences between T1 and T2 were observed in several groups; however, statistically significant within-group changes were limited to selected material–protocol combinations. Therefore, the T1–T2 pattern should be interpreted cautiously and does not allow a generalized conclusion of progressive damage accumulation across all materials and conditions. Studies simulating sequential clinical exposures (impression, brushing, thermocycling) demonstrate that roughness changes become more pronounced with increasing aging intensity and that interactions between material type and aging procedures can be significant [27]. Additionally, evidence indicates that certain bulk-fill composites, particularly those incorporating advanced filler technologies and optimized matrices, may better preserve surface properties (lower Ra, higher VHN), which explains the similarity observed between Groups II and IV in several post hoc comparisons [19,29].

From a clinical perspective, increased surface roughness is relevant as rougher surfaces favor plaque retention and discoloration, while reduced microhardness is associated with increased susceptibility to scratching, wear, and potential marginal degradation. A commonly cited threshold in the literature is Ra ≈ 0.2 μm, above which plaque accumulation may significantly increase; below this value, further reductions in roughness may have limited microbiological impact [30,31,32]. In this context, the higher Ra values observed in subgroup B, particularly for Group III, suggest increased vulnerability under clinical conditions involving repeated acidic exposure combined with masticatory stress. Conversely, the relative stability of Groups II and IV indicates a potentially more robust performance for posterior restorations exposed to variable oral environments, in agreement with reports that certain bulk-fill composites can maintain superior surface properties after aging, depending on composition and protocol [33]. This ranking is broadly consistent with previous studies reporting that composite materials with lower filler loading may show reduced microhardness and greater susceptibility to surface degradation after aging. Accordingly, the lower VHN and higher Ra values observed for Venus Bulk Fill may be explained, at least in part, by its lower filler content, whereas the more stable behavior of Filtek One Bulk Fill and Filtek Supreme Ultra is consistent with their higher filler loading and optimized filler systems [9].

The absence of a statistically significant correlation between VHN and Ra, both globally (rho = −0.009; *p* = 0.958) and within subgroups (A: rho = −0.009; B: rho = 0.052; C: rho = −0.282, *p* = 0.078), suggests that surface topography degradation and reduction in mechanical properties are not necessarily synchronized within this experimental model. This decoupling is plausible, as Ra is primarily influenced by localized phenomena (filler plucking, microcracks, abrasion traces), whereas VHN reflects the integrated response of the superficial and subsurface layers, dependent on matrix composition and crosslink density. Consistently, previous studies indicate that microhardness is predominantly determined by material composition, particularly filler content [20], although inverse correlations between roughness and microhardness have been reported under certain experimental conditions, suggesting that this relationship is highly dependent on the aging environment and material type [18,34]. The negative trend observed in subgroup C warrants further investigation with increased statistical power, as it may indicate an emerging relationship between hydrothermal degradation and surface alterations.

The lack of significant Ra–VHN correlation should therefore be interpreted with caution, as it may also be influenced by the relatively small subgroup size, the use of non-overlapping measurement regions, and the fact that the study was not specifically powered for correlation analysis.

This study has several limitations inherent to the in vitro model: (i) although the same specimens were evaluated at T1 and T2, the use of non-overlapping surface regions and the locally destructive nature of Vickers indentation may limit the direct comparability of repeated measurements and reduce the statistical power for detecting moderate VHN–Ra relationships; (ii) the pH-cycling and thermocycling protocols only partially reproduce the complexity of the oral environment, lacking factors such as biofilm, salivary enzymes, and pellicle formation, although enzymatic degradation of ester-based resins is known to affect long-term mechanical properties [28]; (iii) the use of 2D profilometry and VHN primarily evaluates the surface layer, without complementary microstructural characterization (SEM/EDS/AFM) that could elucidate mechanisms such as filler plucking or silane degradation; and (iv) a single polishing protocol and mechanical loading regimen were applied, whereas clinical behavior may vary depending on technique, antagonist, force magnitude, and dietary conditions. A further limitation is that potential regional variability between the two predefined surface regions cannot be completely excluded, as comparable baseline properties were not confirmed separately for the T1 and T2 measurement areas. Nevertheless, the standardized preparation and polishing procedures were intended to minimize such variability.

A limitation is the absence of baseline measurements before artificial aging; therefore, ΔRa, ΔVHN, or percentage changes could not be calculated, and post-aging differences may partly reflect initial material-dependent properties.

Another limitation is the absence of salivary pellicle and biofilm simulation. In vivo, the acquired pellicle, saliva composition, buffering capacity, and biofilm may modulate acid diffusion, surface softening, and mechanical wear. Therefore, the present pH-cycling model should be interpreted as a simplified accelerated erosive challenge, and direct extrapolation to clinical conditions should be made with caution.

Finally, extending the experimental design to include multiple time points and non-destructive techniques (such as nanoindentation or repeated 3D profilometry) could improve the detection of temporal relationships and VHN–Ra correlations, particularly in subgroup C. Incorporating biological factors and more diverse chemical challenges would allow a more clinically relevant assessment of degradation [35,36,37,38], while combining mechanical and topographical analyses with microstructural investigations and additional parameters could further clarify the mechanisms underlying material differences [21,39].

## 5. Conclusions

Within the limitations of this in vitro study, the following conclusions can be drawn.

The tested aging protocols were associated with significant differences in surface roughness and microhardness at the evaluated post-aging time points. However, because baseline measurements were not performed, the degree of degradation from the initial material condition could not be directly quantified.

The combination of mechanical wear and pH-cycling produced the most pronounced deterioration, indicating a synergistic effect between chemical and mechanical degradation mechanisms, while thermocycling induced moderate changes.

Under the specific conditions of this in vitro study, Filtek One Bulk Fill and Filtek Supreme Ultra showed more favorable post-aging Ra and VHN values under the tested conditions, whereas Venus Bulk Fill showed lower resistance. These findings should not be interpreted as evidence of general clinical superiority, since only one batch and one shade per material were tested and clinical performance may vary depending on patient- and restoration-related factors.

No significant correlation was identified between surface roughness and microhardness, suggesting that these properties may be influenced by partially independent degradation mechanisms.

Clinical relevance

Composite restorations are frequently exposed to combined mechanical and acidic challenges in the oral environment. Under such conditions, materials showing better preservation of surface roughness and Vickers microhardness under combined aging conditions may be preferable in patients with high erosive risk. The findings of the present study highlight the importance of material selection, particularly in patients with high erosive risk or frequent exposure to acidic agents.

## Figures and Tables

**Table 1:** Resin-based materials included in the study and their main characteristics.

Material	Manufacturer	Type	Organic Matrix Composition	Filler Type and Size	Filler Loading (wt%/vol%)
Tetric EvoCeram Bulk Fill	Ivoclar Vivadent	Bulk-fill composite	Bis-GMA, UDMA, Bis-EMA	Barium glass fillers with mean particle sizes of approximately 0.4 and 0.7 μm; overall inorganic filler particle size reported between 40 nm and 3 μm, mean ≈ 550 nm.	~76–77 wt%/~53–55 vol%
Filtek One Bulk Fill Restorative	3M ESPE	Bulk-fill composite	AUDMA, UDMA, DDDMA	Non-agglomerated/non-aggregated 20 nm silica, 4–11 nm zirconia, aggregated zirconia/silica cluster fillers, and ytterbium trifluoride filler; some reports describe ytterbium trifluoride as approximately 100 nm agglomerates.	~76.5 wt%/~58.4 vol%
Venus Bulk Fill	Kulzer	Bulk-fill composite	UDMA-based matrix	Barium-aluminum-fluorosilicate glass, ytterbium fluoride and silicon dioxide fillers; particle size reported approximately 20 nm–5 μm	~65 wt%/~38 vol%
Filtek Supreme Ultra	3M ESPE	Nanofilled composite (control)	Bis-GMA, UDMA, Bis-EMA, TEGDMA	Non-agglomerated/non-aggregated 20 nm silica and 4–11 nm zirconia; aggregated zirconia/silica clusters with average cluster size 0.6–10 μm for dentin/enamel/body shades and 0.6–20 μm for translucent shades	~78.5 wt%/~63.3 vol%

Abbreviations: Bis-GMA—bisphenol A-glycidyl methacrylate; UDMA—urethane dimethacrylate; Bis-EMA—ethoxylated bisphenol A dimethacrylate; TEGDMA—triethylene glycol dimethacrylate; AUDMA—aromatic urethane dimethacrylate; DDDMA—1,12-dodecane dimethacrylate.

**Table 2:** Descriptive statistics of Vickers microhardness according to material, aging protocol, and evaluation time point. Values are presented as median [Q1–Q3].

Aging Protocol	Material	T1 Median [Q1–Q3]	T2 Median [Q1–Q3]
**A**	I	73.57 [70.50–79.11]	71.78 [69.02–74.55]
	II	84.44 [81.19–87.77]	84.85 [82.08–88.64]
	III	67.93 [62.52–70.03]	66.05 [62.97–69.14]
	IV	94.30 [86.36–97.05]	92.20 [89.83–97.52]
**B**	I	65.04 [61.15–68.68]	63.20 [59.22–65.89]
	II	72.61 [68.14–78.31]	71.48 [69.19–73.97]
	III	58.40 [55.66–59.47]	57.63 [55.80–58.28]
	IV	78.84 [73.30–79.89]	76.66 [73.44–78.94]
**C**	I	68.38 [66.72–70.03]	68.00 [65.93–70.14]
	II	80.78 [78.34–83.89]	79.69 [76.83–84.69]
	III	60.94 [58.81–62.16]	60.85 [58.55–62.14]
	IV	84.00 [80.69–86.05]	83.16 [79.86–87.94]

Group codes: I—Tetric EvoCeram Bulk Fill; II—Filtek One Bulk Fill Restorative; III—Venus Bulk Fill; IV—Filtek Supreme Ultra.

**Table 3:** Descriptive statistics of surface roughness (Ra) according to material, aging protocol, and evaluation time point. Values are presented as median [Q1–Q3].

Aging Protocol	Material	T1 Median [Q1–Q3]	T2 Median [Q1–Q3]
A	I	0.16 [0.08–0.20]	0.20 [0.13–0.30]
A	II	0.13 [0.07–0.22]	0.21 [0.16–0.27]
A	III	0.20 [0.12–0.32]	0.23 [0.15–0.34]
A	IV	0.16 [0.15–0.17]	0.19 [0.13–0.24]
B	I	0.24 [0.18–0.28]	0.29 [0.22–0.34]
B	II	0.20 [0.13–0.23]	0.25 [0.19–0.29]
B	III	0.25 [0.18–0.29]	0.24 [0.16–0.31]
B	IV	0.19 [0.14–0.22]	0.24 [0.18–0.29]
C	I	0.19 [0.15–0.24]	0.28 [0.22–0.31]
C	II	0.18 [0.14–0.21]	0.21 [0.17–0.24]
C	III	0.24 [0.16–0.27]	0.28 [0.21–0.31]
C	IV	0.17 [0.13–0.20]	0.19 [0.14–0.22]

Group codes: I—Tetric EvoCeram Bulk Fill; II—Filtek One Bulk Fill Restorative; III—Venus Bulk Fill; IV—Filtek Supreme Ultra.

**Table 4:** Post hoc pairwise comparisons (Dunn–Bonferroni)—Surface roughness (Ra).

Comparison	*p*-Value	Significance	Comparison	*p*-Value	Significance
Subgroups A (T1)			Subgroups A (T2)		
I vs. II	0.082	ns	I vs. II	0.071	ns
I vs. III	0.034	**	I vs. III	0.028	**
I vs. IV	0.041	**	I vs. IV	0.036	**
II vs. III	0.006	**	II vs. III	0.004	**
II vs. IV	0.112	ns	II vs. IV	0.095	ns
III vs. IV	0.002	**	III vs. IV	0.001	**
Subgroups B (T1)			Subgroups B (T2)		
I vs. II	0.049	**	I vs. II	0.041	**
I vs. III	0.003	**	I vs. III	0.001	**
I vs. IV	0.021	**	I vs. IV	0.015	**
II vs. III	0.001	**	II vs. III	0.001	**
II vs. IV	0.038	**	II vs. IV	0.028	**
III vs. IV	0.001	**	III vs. IV	0.001	**
Subgroups C (T1)			Subgroups C (T2)		
I vs. II	0.063	ns	I vs. II	0.052	ns
I vs. III	0.018	**	I vs. III	0.012	**
I vs. IV	0.044	**	I vs. IV	0.038	**
II vs. III	0.003	**	II vs. III	0.002	**
II vs. IV	0.079	ns	II vs. IV	0.061	ns
III vs. IV	0.001	**	III vs. IV	0.001	**

** *p* < 0.05 = statistically significant; ns = not significant. Group codes: I—Tetric EvoCeram Bulk Fill; II—Filtek One Bulk Fill Restorative; III—Venus Bulk Fill; IV—Filtek Supreme Ultra.

**Table 5:** Post hoc pairwise comparisons (Dunn–Bonferroni)—Vickers microhardness.

Comparison	*p*-Value	Significance	Comparison	*p*-Value	Significance
Subgroups A (T1)			Subgroups A (T2)		
I vs. II	0.010	**	I vs. II	0.008	**
I vs. III	0.226	ns	I vs. III	0.198	ns
I vs. IV	0.003	**	I vs. IV	0.002	**
II vs. III	0.001	**	II vs. III	0.001	**
II vs. IV	0.154	ns	II vs. IV	0.132	ns
III vs. IV	0.001	**	III vs. IV	0.001	**
Subgroups B (T1)			Subgroups B (T2)		
I vs. II	0.087	ns	I vs. II	0.065	ns
I vs. III	0.041	**	I vs. III	0.032	**
I vs. IV	0.006	**	I vs. IV	0.004	**
II vs. III	0.002	**	II vs. III	0.001	**
II vs. IV	0.071	ns	II vs. IV	0.059	ns
III vs. IV	0.001	**	III vs. IV	0.001	**
Subgroups C (T1)			Subgroups C (T2)		
I vs. II	0.049	**	I vs. II	0.055	ns
I vs. III	0.072	ns	I vs. III	0.061	ns
I vs. IV	0.008	**	I vs. IV	0.006	**
II vs. III	0.001	**	II vs. III	0.001	**
II vs. IV	0.118	ns	II vs. IV	0.101	ns
III vs. IV	0.001	**	III vs. IV	0.001	**

** *p* < 0.05 = statistically significant; ns = not significant. Group codes: I—Tetric EvoCeram Bulk Fill; II—Filtek One Bulk Fill Restorative; III—Venus Bulk Fill; IV—Filtek Supreme Ultra.

## Data Availability

All data are contained within the article.

## Supplementary Materials

The following supporting information can be downloaded at: https://www.mdpi.com/article/10.3390/dj14060366/s1; Figure S1: Representative profilometry graph for each analyzed group and subgroup in T2.
