# Supplementary figures and images for: Effect of Simulated Oral Aging on Surface Roughness and Microhardness of Bulk-Fill Composite Resins
Source: Dent J (Basel). 2026 Jun 15;14(6):366. doi: 10.3390/dj14060366 (PMC13298019; doi:10.3390/dj14060366)

Figure S1. Representative profilometry graph for each analyzed group and subgroup in T2.

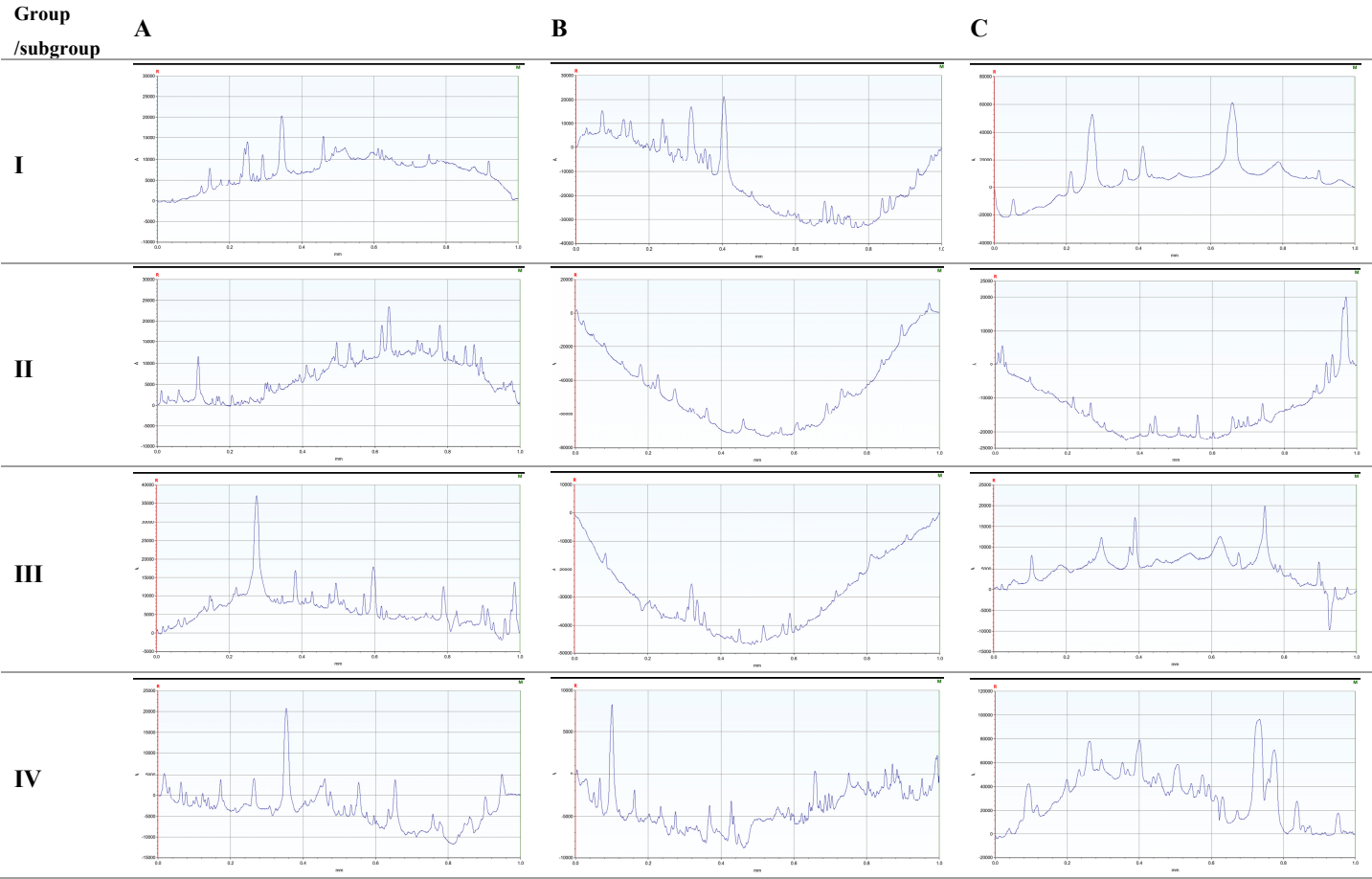

Supplement: Supplementary file 1 [file dentistry-14-00366-s001.zip › dentistry-4329889-supplementary.pdf]
